# Novel Strain of Andes Virus Associated with Fatal Human Infection, Central Bolivia

**DOI:** 10.3201/eid1805.111111

**Published:** 2012-05

**Authors:** Cristhopher D. Cruz, Brett M. Forshey, Efrain Vallejo, Roberto Agudo, Jorge Vargas, David L. Blazes, Carolina Guevara, V. Alberto Laguna-Torres, Eric S. Halsey, Tadeusz J. Kochel

**Affiliations:** US Naval Medical Research Unit 6, Lima, Peru (C.D. Cruz, B.M. Forshey, D.L. Blazes, C. Guevara, V.A. Laguna-Torres, E.S. Halsey);; Servicio Departamental de Salud, Cochabamba, Bolivia (E. Vallejo, R. Agudo);; Centro Nacional de Enfermedades Tropicales, Santa Cruz, Bolivia (J. Vargas);; US Naval Medical Research Center, Silver Spring, Maryland, USA (T.J. Kochel)

**Keywords:** hantavirus, Andes virus, viruses, Bolivia, genetic characterization, zoonoses, human infection, fatal

## Abstract

To better describe the genetic diversity of hantaviruses associated with human illness in South America, we screened blood samples from febrile patients in Chapare Province in central Bolivia during 2008–2009 for recent hantavirus infection. Hantavirus RNA was detected in 3 patients, including 1 who died. Partial RNA sequences of small and medium segments from the 3 patients were most closely related to Andes virus lineages but distinct (<90% nt identity) from reported strains. A survey for IgG against hantaviruses among residents of Chapare Province indicated that 12.2% of the population had past exposure to >1 hantaviruses; the highest prevalence was among agricultural workers. Because of the high level of human exposure to hantavirus strains and the severity of resulting disease, additional studies are warranted to determine the reservoirs, ecologic range, and public health effect of this novel strain of hantavirus.

## Abstract 2

Interventions are needed to reduce human exposure to hantaviruses.

Hantaviruses (family *Bunyaviridae*, genus *Hantavirus*) are trisegmented negative-strand RNA viruses in which the small (S), medium (M), and large (L) genomic segments encode for the nucleocapsid protein (N), 2 envelope glycoproteins (Gn and Gc), and the viral polymerase, respectively. Hantaviruses are maintained in rodent reservoirs, and human exposure typically results from inhalation of aerosols from infectious urine or feces, although human-to-human transmission of Andes virus (ANDV) has also been described (*1*). Human hantavirus infection in South America is often associated with rapid onset of severe disease manifestations, such as respiratory failure and cardiac dysfunction referred to as hantavirus pulmonary syndrome (HPS) and case-fatality rates >50% (*2**,**3*). Despite the public health effects, in most cases of human infection, the precise etiologic agent is not identified. Thus, the extent of genetic diversity and geographic distribution of distinct hantavirus strains is not well understood.

Since the first identification of HPS in 1993, many new hantaviruses have been described throughout North, Central, and South America. Studies of rodent reservoirs in South America have identified an increasingly complex picture of hantavirus diversity and ecology (*2**,**4*). Unique strains of hantavirus have been identified in rodents in Venezuela (*5**,**6*), Peru (*7*), Brazil (*8**–**10*), Argentina (*11**–**13*), Paraguay (*14**,**15*), and Chile (*11**,**16*), many of which have also been associated with human illness. In Bolivia, the first hantavirus identified was Río Mamoré virus (RIOMV), which was isolated from a pygmy rice rat (*Oligoryzomys microtis*) (*17*) but has not been associated with human disease. In 1997, a Laguna Negra virus (LNV) variant was identified in an HPS patient in Chile who had traveled extensively in Bolivia (*18**,**19*). An ecologic assessment of reservoir hosts identified the large vesper mouse (*Calomys callosus*) as reservoir host of LNV in Bolivia (*20*). The association of ANDV (Nort lineage) and Bermejo virus (BMJV) with 2 HPS cases in southern Bolivia in 2000 documented the first human infection by BMJV (*21*).

To further describe the diversity of hantavirus strains associated with human disease in Bolivia, we screened febrile patients reporting to 2 health centers in Chapare Province for serologic and molecular evidence of hantavirus infection. We describe the clinical signs and symptoms and genetic characterization (partial S and M segment) of a novel strain of hantavirus in 3 patients, including 1 who died. In addition, we report results of a survey to determine the prevalence of previous hantavirus exposure in the region.

## Materials and Methods

### Study Site and Human Participant Issues

**Figure 1:**
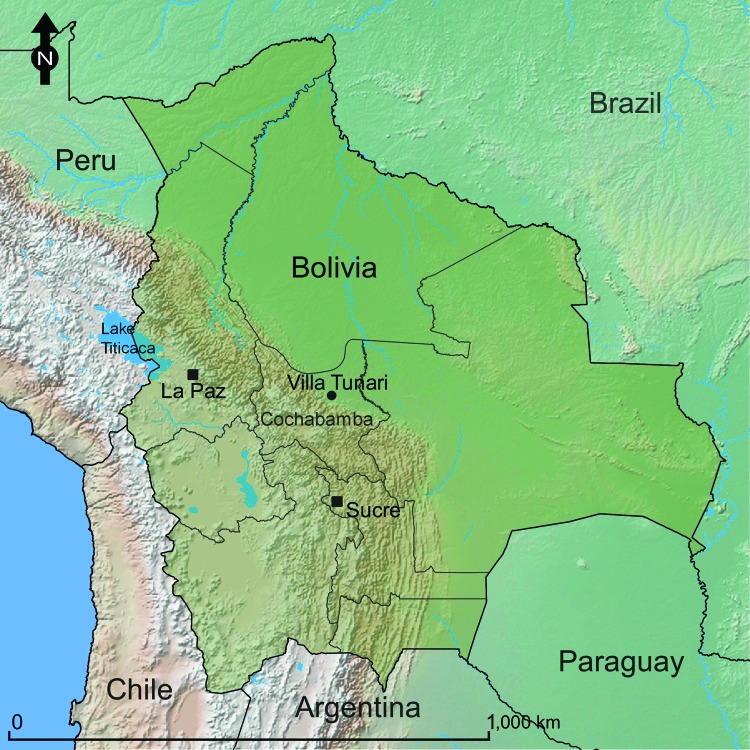
Location of Villa Tunari, Department of Cochabamba, Bolivia, the area where patients with hantavirus infection were recruited. The constitutional (Sucre) and administrative (La Paz) capitals of Bolivia are shown for reference.

Patients were recruited when they reported acute febrile illness (<7 days) at the Hospital San Francisco de Asis or Centro de Salud Eterezama (16°55′S, 65°22′W; 265 m above sea level), located in the Chapare Province of the Department of Cochabamba in central Bolivia (*22*) (Figure 1). Chapare is a rural province with tropical rainforests surrounding the Chapare River, the main waterway of the region. The health centers are located in the towns of Villa Tunari and Eterezama, which had 2,632 and 2,001 inhabitants, respectively, at the time of the 2001 census (*23*).

Study protocols were approved by Servicio Departamental de Salud Santa Cruz, and Colegio Medico de Santa Cruz. Study protocols (NMRCD.2000.0008 and NMRCD.2005.0002) were also approved by the US Naval Medical Research Unit Institutional Review Board in compliance with all US Federal regulations governing the protection of human subjects. Written consent was obtained from patients >18 years of age. For patients <18 years of age, written consent was obtained from a parent or legal guardian. Written assent was also obtained from patients 8–17 years of age.

A survey for prior exposure to arenaviruses and hantaviruses was conducted in Chapare Province during April 25–May 2, 2005, after a reported outbreak of febrile illness and hemorrhagic fever in the region (*24*). Adults (>18 years of age) were invited to participate in the study. Blood samples (10 mL) were collected by venipuncture for screening of antibodies against hantaviruses, and demographic data were collected for risk factor analysis in assorted villages in Chapare Province (Figure 1). Data included age, occupation, self-reported exposure to rodents, house construction materials, and recent health history.

### Serologic Analyses

Serum samples from febrile patients were screened for IgM against ANDV or LNV antigens by ELISA. In brief, 96-well plates were coated with anti-human IgM, human serum samples (1:100 dilution) were added, and plates were incubated for 1 h at 37°C. Wells were subsequently incubated with ANDV or LNV antigen for 1 h at 37°C. Viral antigens were recognized by the addition of hyperimmune mouse ascitic fluid for 1 h at 37°C and incubation with horseradish peroxidase–conjugated anti-mouse IgG for 1 h. Finally, substrate (2,2′-azino-bis-[3-ethylbenzthiazoline-6-sulfonic acid]; Kirkegaard and Perry, Inc., Gaithersburg, MD, USA) was added, and optical density at a wavelength of 405 nm was measured by using a spectrophotometer. Patient serum specimens were also screened for IgM against a panel of arboviral pathogens, including dengue viruses, yellow fever virus, and Venezuelan equine encephalitis virus. Virus culture and identification was attempted in African green monkey Vero cell cultures by indirect immunofluorescence assay and Sin Nombre virus (SNV) polyclonal antibodies, as described for arboviruses (*22*).

For the seroprevalence study, serum samples from healthy participants were screened by indirect ELISA for IgG against SNV antigen (Centers for Disease Control and Prevention, Atlanta, GA, USA). Serum samples were diluted 1:100 and incubated in SNV recombinant antigen–coated wells and then with horseradish peroxidase–conjugated mouse anti-human IgG (1:8,000 dilution). Finally, substrate (2,2′-azino-bis-[3-ethylbenzthiazoline-6-sulfonic acid]) was added, and absorbance was measured at 405 nm with a Dynex ELISA MRX Revelation absorbance reader (Dynex Technologies, Chantilly, VA, USA). Samples with optical densities greater than the mean of 5 negative controls plus 5 SD at a 1:100 dilution were considered positive.

### Molecular Analyses

After serologic screening, RNA was extracted from whole blood and serum samples of patients positive for hantavirus IgM by using the QIAamp Viral RNA Mini Kit (QIAGEN, Valencia, CA, USA). A 1-step reverse transcription PCR (RT-PCR) was performed by using the Access RT-PCR system (Promega, Madison, WI, USA). Nested PCRs were performed by using the FastStart PCR Master (Roche, Indianapolis, IN, USA). Initial screening was performed by using primers specific for the S segment as described (*20*). Additional primers were designed on the basis of preliminary sequences to generate additional S segment coding region sequence (forward: HANSF3 5′-TGGATGTTAATTCCATCGA-3′ and reverse: HANSR4 5′-GATAATGTTTCGTGCTTTCA-3′; forward: HANF0001 TAGTAGTAGACTCCTTGAGAAGCTACT and reverse: HANTASR2 TAGTATGCTCCTTGARAAGC). A 1,287-bp region of the S segment was generated, which included positions 43–1329 of the full-length S segment of ANDV strain Chile R123 (*25*).

For the M segment, RT-PCR and nested PCR were performed by using specific primers (*18*), which generated a 1,330-bp sequence of the M segment that included positions 1678–3007 of the full-length M segment of ANDV strain Chile R123. RT-PCR amplicons were purified by agarose gel electrophoresis and sequenced directly by using the Big Dye Terminator v3.1 Cycle Sequencing Kit on a 3100 Avant-Genetic Analyzer (Applied Biosystems, Foster City, CA, USA).

### Phylogenetic Analysis

S segment and M segment sequences (submitted to GenBank under accession nos. JF750417–JF750422) were compared with sequences from other members of the genus *Hantavirus*, including Puumula virus strain Umea (Genbank accession nos. S segment: AY526219, M segment: AY526218), RIOMV strain HTN-007 (S: FJ532244, M: FJ608550), SNV strain NMH10 (S: L25784, M: L24783), El Moro Canyon virus strain RM97 (S: U11427, M: U26828), Choclo virus (S: DQ285046, M: DQ285047), Caño Delgadito virus (S: DQ285566; M: DQ284451), Pergamino virus (PRGV; S: AF482717, M: AF028028), ANDV strain AH-1 (S: AF324902, M: AF324901), ANDV strain CHI7913 (S: AY228237, M: AY228238), ANDV strain Chile-9717869 (S: AF291702, M: AF291703), Maciel virus strain 13796 (MACV; S: AF482716, M: AF028027), Catacamas virus (CATV; S: DQ256126, M: DQ177347), Paranoa virus (S: EF576661), Oran virus (S: AF482715, M: AF028024), LNV (S: AF005727, M; AF005728), BMJV (S: AF482713, M: AF028025), Lechiguanas virus strain 22819 (S: AF482714, M: AF028022), ANDV strain Hu39694 (S: AF482711, M: AF028023), Playa de Oro virus (S: EF534079, M: EF534082), Neembucu virus (S: DQ345763), Alta Paraguay virus (S: DQ345762), Itapua virus (S: DQ345765), Araraquara virus (ARAV; S: AF307325, M: AF307327), Araucaria virus strain HPR/03–99 (S: AY740630), Jabora virus strain Akp8048 (S: JN232080), Juquitiba virus strain Olfo_777 (S: GU213198), and Castelo dos Sonhos virus (CASV; S: AF307324, M: AF307326).

Sequences were aligned by using ClustalW (www.clustal.org) with manual adjustments, and pairwise genetic distances were calculated by using MEGA4.0 (*26*). For phylogenetic analysis, maximum-likelihood (ML) and Bayesian approaches were used. ML phylogenetic trees were estimated by using PhyML (*27**,**28*) and 100 bootstrap replications to place confidence intervals at nodes. Phylogenetic reconstructions were also conducted in MrBayes version 3.1 (*29**,**30*) under the general time reversible + Γ + proportion invariant model of evolution, with 1 million Markov Chain Monte Carlo generations, and sampling every 100 generations with a burn-in of 25,000. Puumula virus S and M segments were included as outgroups in the phylogenetic reconstructions.

## Results

### Patient Identification

During January 2008–June 2009, serum samples from 372 febrile patients reporting to clinics in Chapare Province, Bolivia (Figure 1) were tested for serologic evidence of recent infection by >1 hantaviruses. Of these 372 patients, 199 (53.5%) were male patients with a median age of 31 years (range 7–95 years). IgM against ANDV (n = 8) or LNV (n = 1) antigen was identified in acute-phase or convalescent-phase samples from 9 (2.4%) patients. No evidence of recent arbovirus infection was detected in these samples. Of the 9 patients with IgM against hantaviruses, 7 (77.8%) were male patients with a median age of 32 years (range 15–49 years). Three of the 9 patients were positive for hantavirus RNA.

Patient 1 (FVB0554) was an 18-year-old man (student) from the town of Pedro Domingo Murillo, Bolivia, who came to Hospital San Francisco de Asis in January 2008 He reported 7 days of fevers, chills, and malaise. Other symptoms included oliguria, arthralgias, myalgias, bone pain, headache, and retroocular pain. Gastrointestinal (abdominal pain, diarrhea, nausea, emesis, and icterus) and respiratory (cough, dyspnea, and cyanosis) manifestations were also prominent. The patient died the next day. IgM against LNV antigen (reciprocal titer 1,600) was detected in a serum sample collected before his death.

Patient 2 (FVB0640) was a 27-year-old man (agricultural worker) from Samuzabety, Bolivia, who came to Hospital San Francisco de Asis in March 2008. The patient had a temperature of 39.9°C and reported 8 days of fever, chills, and malaise. Other symptoms included cough, arthralgias, myalgias, bone pain, headache, and retroocular pain. On examination, multiple cutaneous manifestations were noted, including petechiae, purpura, a maculopapular rash, and a diffuse erythematous rash. The patient was hospitalized for 4 days and recovered. IgM against ANDV was detected in an acute-phase serum sample (reciprocal titer 6,400); no convalescent-phase sample was obtained.

Patient 3 (FVB0799) was a 49-year-old man (farmer) from Flor de San Pedro, Bolivia, who came to Hospital San Francisco de Asis in June 2009. He reported 4 days of fever, chills, and malaise. Other symptoms included arthralgias, myalgias, bone pain, abdominal pain, headache, cough and dyspnea. The patient survived. IgM against ANDV was detected in an acute-phase serum sample (reciprocal titer 6,400); no convalescent-phase sample was available for additional analysis.

### Molecular Analyses

Viral sequences generated from samples from the 3 patients were highly conserved over the gene regions analyzed; >99.6% pairwise nucleotide identity in the S segment (3–5-nt differences) and >99.2% pairwise nucleotide identity in the M segment (1–10-nt differences). Nucleotide sequences were compared with those of hantavirus strains available in GenBank (Table 1). In pairwise comparisons of S segment gene sequences, we observed the highest identity with CASV (*31*), which showed 89.3% identity at the nucleotide level and 98.6% identity at the amino acid level, although only limited sequence (643 nt) was available for comparison. In comparison with other Western Hemisphere hantaviruses for which more extensive sequences were available (1,287 nt) 75.8%–84.1% nucleotide sequence identity and 85.3%–97.7% amino acid identity were observed, and the highest similarity was with members of the species *Andes virus* (Table 1).

**Table 1:** Percent pairwise nucleotide and amino acid identity between select Western Hemisphere hantaviruses and virus sequences amplified from patients from central Bolivia*

Virus strain	Country	S segment (1,287 bp)			M segment (1,330 bp)	
		Nucleotide	Amino acid		Nucleotide	Amino acid
PRGV	Argentina	81.4	94.6		80.8	93.0
ANDV AH1	Argentina	83.5	96.0		81.7	93.9
ANDV Hu39694	Argentina	82.0	97.4		81.7	95.3
MACV	Argentina	81.7	94.2		80.2	91.4
BMJV	Argentina	83.7	97.7		80.2	93.9
LECV	Argentina	84.1	97.4		81.1	95.0
ORNV	Argentina	83.5	97.4		80.3	95.3
CASV†‡	Brazil	89.3	98.6		83.3	95.1
PARV	Brazil	82.9	95.3		NA	NA
ARAV§	Brazil	84.0	94.9		79.5	93.2
JABV	Brazil	77.3	88.6		NA	NA
ARCV	Brazil	82.2	95.8		NA	NA
ANDV 9717869	Chile	83.5	96.0		80.7	93.7
ANDV CHI7913	Chile	82.7	95.6		81.1	92.8
CATV	Honduras	76.9	88.1		76.0	86.2
PDOV	Mexico	77.4	87.4		75.8	85.4
CHOV	Panama	78.9	89.3		77.8	88.0
NEMV	Paraguay	84.9	97.0		NA	NA
ALPV	Paraguay	80.3	89.3		NA	NA
ITAPV	Paraguay	81.7	95.8		NA	NA
JUQV‡	Paraguay	82.5	95.5		NA	NA
LNV	Paraguay	79.4	90.2		79.2	90.5
RIOMV	Peru	80.1	90.0		80.6	91.4
SNV NMH10	USA	76.5	87.2		76.0	86.2
ELMCV RM97	USA	76.8	83.9		73.8	82.8
MAPV	Venezuela	79.6	91.1		77.8	89.8
CADV	Venezuela	75.8	85.3		74.3	83.1

*S, small; M, medium; PRGV, Pergamino virus; ANDV, Andes virus; MACV, Maciel virus; BMJV, Bermejo virus; LECV, Lechiguanas virus; ORNV, Oran virus; CASV, Castelo dos Sonhos virus; PARV, Paranoa virus; ; NA, sufficient sequence not available for comparison; ARAV, Araraquara virus; JABV, Jabora virus; ARCV, Araucaria virus; CATV, Catacamas virus; PDOV, El Moro Canyon virus; CHOV, Choclo virus; NEMV, Neembucu virus; ALPV, Alta Paraguay virus; ITAPV, Itaporanga virus; JUQV, Juquitiba virus; LNV, Laguna Negra virus; RIOMV, Río Mamoré virus; SNV, Sin Nombre virus; ELMCV, El Moro Canyon virus; MAPV, Maporal virus; CADV, Caño Delgadito virus. †S segment sequence comparison was limited to the homologous 999 bp (JUQV) or 643 bp (CASV) available from GenBank. ‡M segment sequence comparison was limited to the homologous 1,246 bp available from GenBank.

In pairwise comparisons of M segment gene sequences, the highest nucleotide identity (83.3%) was observed in comparison with CASV. Similar amino acid identities were observed with CASV (95.1%), Oran virus (95.3%), Lechiguanas virus (95.0%), and ANDV Hu39694 (95.3%) (Table 1). Viral sequences amplified from patient samples were more distantly related to LNV, Caño Delgadito virus, and Maporal virus; all showed <80% pairwise identity at the nucleotide level and <90% pairwise identity at the amino acid level (Table 1).

**Figure 2:**
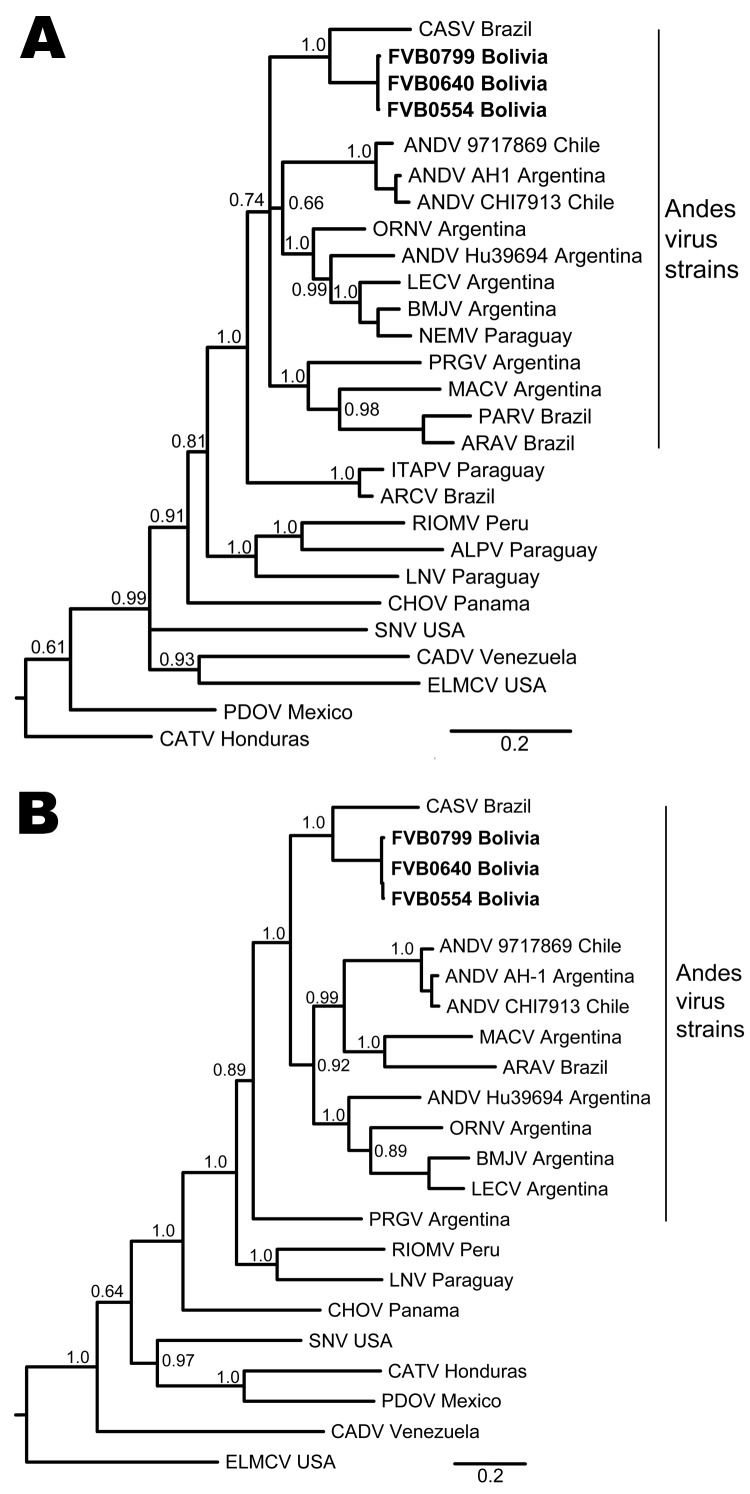
Phylogenetic analysis of hantaviruses from the Western Hemisphere on the basis of partial A) small and B) medium segments. Novel strains described in this study are indicated in **boldface**. Depicted phylogenetic reconstructions are based on Bayesian inference conducted in MrBayes (*29**,**30*). Posterior probabilities are indicated at relevant nodes. Scale bar indicates nucleotide sequence divergence. CASV, Castelo dos Sonhos virus; ANDV, Andes virus; ORNV, Oran virus; BMJV, Bermejo virus; LECV, Lechiguanas virus; BMJC, Bermejo virus; NEMV, Neembucu virus; PRGV, Pergamino virus; MACV, Maciel virus; PARV, Paranoa virus; ARAV, Araraquara virus; ITAPV, Itaporanga virus; ARCV, Araucaria virus; RIOMV, Río Mamoré virus; ALPV, Alta Paraguay virus; LNV, Laguna Negra virus; CHOV, Choclo virus; SNV, Sin Nombre virus; CADV, Caño Delgadito virus; ELMCV, El Moro Canyon virus; PDOV, El Moro Canyon virus; CATV, Catacamas virus.

To further explore genetic relationships between the novel viral sequences and previously described hantaviruses, we conducted ML and Bayesian analyses on the basis of S segment and M segment nucleotide sequences. Similar results were obtained for ML and Bayesian approaches (Figure 2). Viral sequences derived from patient samples grouped with other strains of ANDV (www.ncbi.nlm.nih.gov/ICTVdb/index.htm); formed a clade with ARAV, MACV, PRGV, and other ANDV strains; and formed a subclade with CASV (Figure 2). Similar tree topologies for other strains of ANDV were obtained on the basis of analysis of S segment and M segment sequences. Genetic differences between CASV and the novel sequences were well supported by posterior probabilities (Figure 2) and ML bootstrap values.

### Prevalence of IgG against Hantaviruses among Humans in the Chapare Region

To determine the extent of human exposure to hantaviruses in the region, we screened serum samples from residents of villages in Chapare Province for IgG against SNV antigen. A total of 500 participants >18 years of age residing in villages in the region were enrolled during April 25–May 2, 2005 (Table 2). Participants had a median age of 31 years (range 18–99 years); 54.9% were women (Table 2).

**Table 2:** Characteristics of patients tested for IgG against Sin Nombre virus, central Bolivia*

Characteristic	No. positive/no. tested (%)
Sex	
M	28/224 (12.5)
F	32/273 (11.7)
Age, y	
18–30	28/244 (11.5)
31–50	28/207 (13.5)
>50	4/43 (9.3)
Occupation	
Agricultural worker	25/167 (15.0)
Housewife	26/193 (13.5)
Student/teacher	3/57 (5.3)
Health professional	0/20 (0)
Other/unknown	7/62 (11.3)
Village	
Eterazama	13/116 (11.2)
Isinuta	6/71 (8.5)
Primero de Mayo	1/ 20 (5.0)
Samuzabety	13/70 (18.6)
San Gabriel	5/ 29 (17.2)
San Julian	2/24 (8.3)
Urkupina	2/22 (9.1)
Other	19/148 (12.8)
Total	61/500 (12.2)

*Complete demographic data were not available for all participants.

Sixty-one (12.2%; 95% CI 9.3%–15.1%) had IgG against SNV antigen (Table 2), and the highest prevalences were in the towns of Samuzabety (18.6%) and San Gabriel (17.2%). No differences were observed between sexes or among different age groups (Table 2). The highest prevalence of IgG against SNV was among agricultural workers (15.0%) and housewives (13.5%) (Table 2). No differences in seropositivity were observed for participants with differing house construction materials or quality.

## Discussion

We demonstrated the association of a novel genotype of ANDV with fatal human infection in central Bolivia and extended the known genetic diversity of hantaviruses circulating in South America. One fatal case occurred among the 3 patients described, which was consistent with high mortality rates observed with infections with ANDV lineages in neighboring Brazil and Argentina (*3*). The International Committee on Taxonomy of Viruses has provided guidelines for the demarcation of hantaviruses (www.ictvdb.org/Ictv/index.htm), which include a >7% difference in amino acid identity in comparison with previously identified complete S segment and M segment gene sequences, a >4-fold difference in results of 2-way cross-neutralization tests, and occupation of a unique ecologic niche, such as a different primary rodent reservoir. As with other hantavirus strains, attempts to isolate virus in tissue culture were unsuccessful; thus, cross-neutralization studies could not be conducted. Genetic criteria, amino acid and nucleotide comparisons, and phylogenetic analysis clearly support inclusion of this strain in the species *Andes virus*.

No guidelines have been proposed for demarcation of viruses below the species level, and there does not appear to be consensus on the designation of novel genotypes. We observed the highest identity with CASV, which has been associated with human illness near the border of the Mato Grosso and Pará States of Brazil (*31**,**32*), ≈1,500 km from the Chapare study sites. We observed ≈11% and 17% divergence at the nucleotide level for the S segment and M segment, respectively, in comparison with CASV. However, the true difference between the strains might be underestimated because higher nucleotide and amino acid conservation was observed among ANDV strains overall in the limited S region available for comparison (14%), relative to other genome regions (16%). No other hantavirus was found to be <15% divergent at the nucleotide level in the S segment or <18% divergent in the M segment. If one considers that the strains identified in our study segregate with other strains of ANDV but are genetically distinct, we provisionally propose to name this novel genotype Tunari virus (TUNV), after the town of Villa Tunari, where all 3 patients sought medical attention.

On the basis of data in this report, we found that human hantavirus exposure is common in the Chapare Province. Including the 3 TUNV cases, in 2008 and 2009, >2% of febrile cases analyzed had evidence of recent hantavirus infection, which is consistent with the 4% of febrile cases reported for the region in 2005 and 2006 (*33*). In addition, the 2005 serosurvey of healthy persons indicated that a high percentage (≈12%) of adults had evidence of exposure to >1 hantaviruses at some time in their lives.

The extent of exposure we found is similar to that reported in neighboring Brazil, which was 13.3% in Maranhao State in northeastern Brazil (*34*) and 14.6% in southern Brazil (*35*), and in northern Argentina, which was 6.5%–17.1%, depending on the population studied (*13**,**36*). Occupational exposure appears to be a risk factor because the highest antibody prevalence and 2 of 3 TUNV cases were identified among agricultural workers. We did not observe an age-dependent increase in antibody prevalence among adults sampled, a finding also reported in southern Brazil (*35*). There are several possible explanations for this observation, including relatively recent emergence of hantaviruses in the region, high mortality rates among infected persons, and preponderance of risk for exposure during early adulthood.

Broad antigenic cross-reactivity that prevents discrimination among diverse hantavirus groups is 1 of the major limitations of ELISA-based serologic studies, whether used in screening febrile patients for IgM against hantaviruses or healthy persons for IgG against hantaviruses. Co-circulation of heterologous hantaviruses has been described in Bolivia in rodent reservoirs and in ill persons. RIOMV has been identified in the pigmy rice rat (*Oligoryzomys microtis*) in Bolivia (*17*). In 2000, HPS cases associated with BMJV and ANDV strain Nort were identified along the southern border of Bolivia with Argentina (*21*). LNV had been amplified from an HPS patient in Chile with recent travel history to Bolivia (*19*). In addition to these cases are many additional suspected cases of HPS in Bolivia for which no specific virus was identified. Of the 246 reported cases from 2007 through 2010, a total of 74 occurred in the Department of Cochabamba (*37*). Future studies with more specific serologic assays are necessary to determine the true extent of TUNV circulation in this population.

In this study, we made no effort to incriminate the reservoir host for TUNV. The only hantavirus reservoir identified in South America is rodents of the subfamily *Sigmodontinae*. *Oligoryzomys* spp. rodents appear to be the principal reservoirs for most ANDV variants, including CASV (*32**,**38*). In addition to *Oligoryzomys* spp. rodents, ANDV variants have been identified in *Akodon* spp. (PRGV), *Necromys* spp. (MACV and ARAV), and *Bolomys* spp. (MACV) rodents. Potential reservoir species are abundant in Bolivia, including *Oligoryzomys* spp., *Akodon* spp., and *Calomys* spp. (LNV) rodents. Increased rodent population density has been associated with the emergence of hantavirus infection in humans (*4*). Therefore identifying the TUNV reservoir host and understanding its ecology could lead to interventions for reducing human exposure.
